# Common genetic control for grain filling duration and kernel weight in grain sorghum

**DOI:** 10.1007/s00122-026-05409-0

**Published:** 2026-10-12

**Authors:** Daniel Otwani, Colleen Hunt, Alan Cruickshank, Yongfu Tao, Anna M. G. Koltunow, David Jordan, Emma Mace

**Affiliations:** 1 Queensland Alliance for Agriculture and Food Innovation (QAAFI), The University of Queensland, Hermitage Research Facility, Warwick, QLD Australia; 2 Department of Primary Industries (DPI), Queensland Government, Hermitage Research Facility, Warwick, QLD Australia; 3 Queensland Alliance for Agriculture and Food Innovation (QAAFI), The University of Queensland, St Lucia, QLD Australia; 4 Australian Research Council Centre of Excellence for Plant Success in Nature and Agriculture, The University of Queensland, St Lucia, Brisbane, QLD 4067 Australia; 5 Agricultural Genomics Institute at Shenzhen, Chinese Academy of Agricultural Sciences, Shenzhen, 518120 Guangdong China

## Abstract

***Key message*:**

The genetic control of GFD in sorghum closely overlaps with that of grain size, is influenced by both pre- and post-anthesis processes, offering new opportunities for yield improvement through targeted breeding and inter-racial trait introgression.

**Abstract:**

The duration of the grain filling period has been associated with yield increases in cereals including maize and sorghum. The genetic control of grain filling duration (GFD) is however not known in sorghum. This study explored the genetic variation and extent of genetic control for GFD in a diverse panel of sorghum genotypes (n=904), in three environments across two years. A genome wide association analysis revealed 86 QTLs, 46 of which collocated with 54 previously reported grain size candidate genes in sorghum, indicating a significant enrichment. Single marker analysis revealed that genomic regions associated with GFD were similarly associated with grain size. Interestingly, in silico expression analysis of candidate genes associated with GFD revealed that GFD could be associated with processes that happen both before and after anthesis contrary to the understanding that GFD was primarily associated with processes that happen post anthesis. Haplotype analysis of *SbGS3* resolved 8 haplotypes associated with grain filling duration, 2 of which were exclusive to the Guinea and Asian durra racial groups revealing opportunities for trait introgression across sorghum racial groups. These results indicate considerable opportunity to increase grain yield in sorghum, by selecting for longer GFD and diverse inter racial crosses to improve the genetic diversity for GFD in sorghum. Sorghum breeders will find application of these results in diversifying trait selection to optimise yields in changing environments.

**Supplementary Information:**

The online version contains supplementary material available at https://doi.org/10.1007/s00122-026-05409-0.

## Introduction

Grain filling, defined as the period between anthesis and physiological maturity, plays a critical role in determining maximum grain size (Egli 2006). The rate and duration of grain filling together determine grain size, which, when combined with grain number per unit area, determines grain yield (Boyles et al. 2016; Otwani et al. 2024; Van Oosterom and Hammer 2008). To date, genetic gain for yield in sorghum has been achieved primarily through changes in grain number (Demarco et al. 2023), with similar trends being observed in other cereals like maize (Russell 1991) and rice (Khush 1995). Recent studies on maize yield improvements over five decades (Fernández et al. 2022; Xing et al. 2023) reveal that increases in grain weight among hybrids of different eras were largely due to extended GFD, suggesting that targeting this trait could enhance yield gains in cereals. Otwani et al. (2025) report potential yield benefits in sorghum through increasing GFD, suggesting that exploiting GFD has potential to overcome (Gambín and Borrás 2012; Yang et al. 2010) the negative correlation between grain size and number (Sadras 2007) while breeding for increased yield.

There is limited research on the extent and genetic control of variation for GFD in sorghum. Yang et al. (2009), observed that pre-anthesis ovary volume is varied across sorghum genotypes and was correlated with GFD and grain size on a set of three genotypes. This result is consistent with a study by Tao et al. (2021) indicating that grain size is limited more by the genetic potential of grain size set pre-flowering rather than assimilate supply suggesting that there may be potential to exploit genetic variation in grain size to increase grain size in sorghum. To date more than 100 quantitative trait loci (QTLs) have been identified in sorghum for grain size and weight across diverse studies (Boyles et al. 2017; Han et al. 2015; Paterson et al. 1995; Tao et al. 2018, 2020), some of which are in common with other grain size related traits like grain length, width, volume and grain thickness. Despite the QTLs reported for grain size, only a few predicted candidate genes have been identified in sorghum. *Sobic.001G341700* is predicted to be the causative gene for *qTGW1a* which acts as a negative regulator of grain size in sorghum (Zou et al. 2020) homologous to *GS3* in rice. Further, studies to explore other traits associated with grain size and their genetic control in sorghum are limited. To our knowledge, no study is available on the genetic control of GFD in sorghum.

Some studies in maize and rice have reported some predicted genes and transcription factors responsible for grain filling. Three transcription factors, *NAKED ENDOSPERM1* (*NKD1*)*, NKD2* and *OPAQUE2* (*O2*) have been reported to function in endosperm cellular development and promoting biosynthesis and storage of starch, proteins and lipids in the developing maize seed (Wu et al. 2024). The transactivation by *O2* of sucrose synthase1 (*Sus1*) and *Sus2* mediates endosperm filling in maize (Deng et al. 2020), and transactivation by *O2* of a *DELLA*-like transcriptional regulator, *ZmGRAS11* mediates synergistic endosperm enlargement with grain filling (Li et al. 2021). In rice, a prolonged GFD mutant 1 (*gfd1*), show a longer GFD, less grain number per panicle and bigger grain size. *GFD1* interacts with sugar transporters *OsSWEET4* and *OsSUT2* to mediate GFD and grain size respectively and with both *OsSWEET4* and *OsSUT2* to regulate grain number (Sun et al. 2023). Some predicted gene models have been identified in sorghum through stage specific gene expression analysis including grain filling period (Costes et al. 2024; Cruet-Burgos and Rhodes 2023; Jain et al. 2024). For instance, *Sobic.002G367600*, an orthologue of *CYP78A13* in rice, a regulator of size balance between embryo and endosperm (Nagasawa et al. 2013) has been reported to be highly expressed during grain filling in sorghum (Jain et al. 2024). Carbohydrate metabolism genes have been shown to be highly expressed during the grain filling period like the *waxy* (*wx Sobic. 010G022600*) (Jain et al. 2024) and *SUGARY* (*SbSu*;*Sobic.007G204600*) which has a regulatory role in starch synthesis (Hashimoto et al. 2023). Similarly in other cereals, amylase inhibitors have been reported to accumulate from one week after anthesis through to physiological maturity in wheat (Call et al. 2021) and rice (Hakata et al. 2012) and function to improve grain quality by repressing starch degradation suggesting that these functions may be conserved in cereals.

Several studies in sorghum have attempted to explore the genotypic diversity and physiology of GFD and its association with yield (Done 1986; Gambín and Borrás 2012; Otwani et al. 2025; Schaffer 1981). While these studies are pivotal in establishing a potential link between an extended GFD and yield, the exploitable genetic variation for GFD in diverse sorghum genotypes remains to be studied at depth, so is the genetic control and association with other yield determining traits in sorghum. For instance, grain size genetics is widely studied in sorghum (Tao et al. 2017, 2018, 2020, 2021), but association with GFD is lacking despite the established association in other cereals (Fernández et al. 2022; Williams et al. 2024).

In the present study, we hypothesise (1) that genetic variation for GFD is available in sorghum diverse germplasm, (2) that the GFD could be extended independent of flowering time and maturity, and (3) that genetic/genomic controls of GFD could be dissected by examining the onset and progression of grain filling. We applied a population genetics approach to investigate the natural variation of GFD within a diverse sorghum panel that includes representatives from all the sorghum racial groups described in (Doggett 1988). Through genome-wide association analysis, we identified genomic regions and candidate genes that may be involved in regulating GFD.

## Material and methods

### Plant materials and experiments

The sorghum diversity panel (DP, n = 904), previously described by (Otwani et al. 2025; Tao et al. 2020), was used in the current study. Three experiments were planted, two at the Hermitage Research Facility (HRF), Warwick, Queensland, Australia (28˚ 12ʹ S, 152˚ 5ʹ E, 470 m above sea level) in November 2020 and December 2021, and the third was planted at Gatton Research Facility (GAT), Gatton, Queensland, Australia, (27˚ 33ʹ S, 152˚ 20ʹ E, 94 m above sea level) in February 2021. At HRF, 881 DP genotypes were planted in a row column design with partial replication where 30% of the genotypes were replicated two or more times while the remaining 70% were in single plots in 2020/21 season (HRF1) and a fully replicated trial in 2021/22 season HRF2. At GAT, a total of 609 DP lines were planted in a fully replicated trial of two replications in a row column design. All the trials were planted during the Australian summer growing season in single row plots 4 m long. Standard agronomic practices were employed in the trial management to ensure adequate nutrition and pest and weed control. Overall, the experiments had 598 DP genotypes in common.

### Phenotypic evaluation

Single plants of each genotype were tagged in each plot at the time of head exsertion prior to onset of flowering. All measurements for timing of flowering and maturity were recorded on the same tagged plant. Flowering time (DTF) was recorded as the date when the first anthers become visible at the tip of the panicle. The tagged plant was monitored throughout the season, and the date of physiological maturity (DTM) was recorded as the date when a sampled grain from the tip of the panicle first showed the abscission layer (black layer) at the point of connection of the grain. Plant height was measured at HRF2 by selecting one representative plant at random from the plot and measuring the distance from the base of the plant to the tip of the panicle at physiological maturity. Single panicles were harvested at HRF2, dried in a tobacco barn until constant moisture, threshed, and cleaned before grains per panicle, and thousand kernel weight (TKW) were measured using an automatic seed counter and weighing machine (Ball Coleman Gen3 seed counter). Daily weather data was recorded using a portable weather station placed within the trial to record daily maximum and minimum air temperatures for the duration of the experiment. The temperature data was used in the estimation of thermal time accumulated for respective growth and development phases as described in (Hammer and Muchow 1994), the base temperature for GFD was 5.7 degree Celsius. Overall, the trial at Gatton experienced lower temperatures during anthesis and post-anthesis in the grain filling period.

### Statistical analyses

Linear mixed models were fitted as a multi-environment trial (MET) analysis and used to predict Best Linear Unbiased Predictions (BLUPs). The MET model was also used to estimate correlations between the study traits. All the traits were analysed using a linear mixed model and the residuals assessed for normality.

The standard representation of a linear mixed model is given by;

1 *y = Xτ + Zu + e*

where y is the vector of observations with the sites stacked, X is the design matrix for fixed effects, τ is the vector of fixed effects, Z is the design matrix for random effects, u is the vector of random effects which has a normal distribution with mean 0 and variance G (u~N (0, G)), with fixed and random spatial effects included as necessary (see Supplementary Table 1) (Gilmour et al. 1997) and e is the vector of residuals e~N (0, R).

All the sites had significant autoregression correlations in both the column and row directions and a random effect. HRF1 had a spline effect in the column direction for both the time to flowering and duration to maturity traits but not for the grain filling duration, since the site was uneven. HRF2 had a linear trend in the column direction.

Best Linear Unbiased Estimates (BLUEs) were estimated by including the genotypes as fixed effects in model (1) (contains a main effect for genotypes at Gatton only) while BLUPs were predicted from model (1) where site x genotype was included as a random effect. The variance–covariance matrix for the site by genotype interaction (GxE) was fitted using a general correlation structure with heterogenous variances (corgh) in ASReml-R. This structure allows for a different genetic variance for each site and different correlations for each pair of sites. Different models were fitted separately for each trait, with random and fixed terms included as necessary per site, see Supplementary *Table *1. Broad sense heritability was estimated per site using the generalised heritability method as described in (Cullis et al. 2006).

All analyses were conducted in R (RCoreTeam 2024) environment version 4.04, the package ASReml-R (TheVSNiTeam 2023) was used to fit all models and the packages ggplot2 (Wickham 2016) and GGally (Schloerke et al. 2021) was used in visualising the figures.

### Molecular marker data

Procedures for genomic DNA extraction and sequence data construction were described previously (Mace et al. 2019; Tao et al. 2018). In total, 726,309 SNPs were identified and aligned to sorghum genome assembly version v3.1.1. This diversity panel was resequenced using DArTreseq technology (Edet et al. 2018), and conducted by Diversity Arrays Technology Pty Ltd https://www.diversityarrays.com/technology-and-resources/dartreseq/. After trimming adapters and filtering low-quality reads, the clean reads were mapped to the reference genome BTx623 (v3.1.1) (McCormick et al. 2018) with Burrows-Wheeler Alignment software (version 0.7.8) using the *mem* command (Li and Durbin 2009) to call SNPs.

### GFD across sorghum racial groups

The 881 DP genotypes had been allocated racial group membership based on a population structure analysis as described in (Tao et al. 2020), with a threshold of 70% genetic identity for a genotype to be allocated to a given racial group. The racial variation was analysed across each trial independently and across all trials together. Across trial data is presented.

### GWAS analysis and QTL identification

The Fixed and random model Circulating Probability Unification (FarmCPU) software described in (Liu et al. 2016) was used for GWAS analysis while accounting for population structure using principal component analysis (PCA). BLUPs were used as the inputs to the GWAS model, performed separately for each of the three sites. 726,309 SNPs (minor allele frequency > 0.01) after imputation and filtering were realised and used for the GWAS analysis. For potential QTL identification, the package simple ℳ (Gao et al. 2008) was used to determine an estimate of the number of independent tests which was then used in the determination of the cut off P-value for the selection of effective SNPs (Significant and Suggestive SNPs). Thus, a cut off P-value of 1.53e−07 for HRF1 and HRF2 analysis and 1.65e−07 for GAT analysis was identified for significant SNPs and P-value of < 9.79e−05 for suggestive SNPs. SNPs from the three environments were collated and those sitting within 1 cM of each other within a chromosome were in the same QTL region. The QTLs were designated with letters QGFD, Q for QTL and GFD the trait and consecutive numbers starting with the chromosome number followed by a decimal point and numbers showing the number of QTLs within the chromosome (QGFD1.1 would be the first QTL in chromosome one). Further, post GWAS analyses were conducted to compare coincidence of GFD QTLs with previously identified QTLs for grain size and other grain related traits in sorghum. To compare the overlap of GFD QTLs with previously identified grain size-related QTLs, we reviewed several studies: Takanashi et al. (2021) with 213 RILs, Zou et al. (2020) with 244 RILs, Tao et al. (2020) with 837 diversity panel lines and 1,421 BC-NAM lines, and,(Tao et al. 2021) which involved manipulating assimilate supply. Further comparisons between detected QTLs in this study and previously reported sorghum QTLs were performed using the QTL Atlas (Mace et al. 2019) (https://aussorgm.org.au/sorghum-qtl-atlas/). To search for sorghum orthologs of rice or maize responsible genes, we used the BLASTP program in the Phytozome database (https://phytozome-next.jgi.doe.gov/). A priori candidate genes were further explored from previous studies that identified candidate genes associated with grain size, starch and protein content (Jain et al. 2024; Tao et al. 2018, 2021, 2020) and genes expressed from pollination to maturity in grain sorghum (Jain et al. 2024). A 1 centimorgan (cM) window was used to identify collocation of the GFD QTLs with the candidate genes. The 1 cM window used in this study was based on the sorghum consensus genetic map published by Mace et al. (2009). The conversion between genetic and physical positions was also according to this consensus map. The criteria applied here follow previous studies using the same population (Tao et al. 2021, 2020).

### Single marker analysis

All the effective SNPs identified for GFD across the three locations from the GWAS were collated and used for single marker analysis. The SNP effects on GFD were analysed in a linear mixed model framework with all significant SNPs included simultaneously as fixed effects in the model with GFD first as the response, then the process was repeated with TKW as the response. The random terms included genotypes and marker data in a variance model to account for kinship and structure within the genotypes. The residual term was an autoregressive model in the column and row directions. The model equation is described below;

$$\mathbf{Y} = \mathbf{X}\boldsymbol{\tau} + \mathbf{Z}\mathbf{u} + \mathbf{e}$$

where y is the vector of observations, X is the design matrix for fixed effects, τ is the vector of fixed effects, Z is the design matrix for random effects, u is the vector of random effects which has a normal distribution with mean 0 and variance G (u~N (0, G)), with fixed and random spatial effects included as necessary (Gilmour et al. 1997) and e is the vector of residuals e~N (0, R). For potential QTL identification, the package simple ℳ (Gao et al. 2008) was used to determine an estimate of the number of independent tests which was then used in the determination of the cut off P-value for the selection of effective SNPs. The P-value used was 0.000236967 for significant SNPs and 0.004739336 for suggestive SNPs.

### Haplotype analysis for *Sobic.001G341700*

While several candidate genes were identified in the current study, *Sobic.001G341700* (*SbGS3*) was specifically used as an example for haplotype analysis because it is a validated gene in sorghum (Zou et al. 2020) and is associated with grain size in both sorghum (Ananda et al. 2023; Zou et al. 2020), maize (Li et al. 2010) and rice (Fan et al. 2006). The haplotype analysis of the qTGW1a (*Sobic.001G341700*) an orthologue of *GS3* in rice in the sorghum diversity panel were performed using the SNPs data on genomic sequence using *Sorghum bicolor* genome v3.1.1 for the entire qTGW1a (Zou et al. 2020). The package vcfR (Knaus and Grünwald 2017) was used for the initial extraction of the genotypic information from the vcf file. The packages adegenet (Jombart 2008) (version 2.1.10) was used to convert data into a format suitable for further population analysis and clustering, while ade4 (Dray and Dufour 2007) was used for generation of principal components (PCs). The PCs summerised the genetic variation and clustered the genotypes into gene specific haplotype groups that were then associated with the various racial groups. The resulting haplotypes for the gene were visualized using the ggplot2 package (Wickham 2016).

## Results

### Phenotypic variation in GFD

GFD ranged from 400 to 680-degree days with the means across the genotypes for each experiment being 510, 506 and 521-degree days for GAT, HRF1 and HRF2 respectively. Appreciable genetic variation for GFD was observed, with moderate broad sense heritability estimates ranging between 41 and 61%. Genetic correlations between sites ranged from 0.45 to 0.86 with HRF1 and HRF2 having a stronger genetic correlation and a less strong genetic correlation reported between HRF2 and GAT. A comparison of GFD across the sorghum genotypes as defined by racial grouping (Otwani et al. 2025) showed that race Guinea had on average a longer GFD in comparison to all the other racial groups in each site and from the combined analysis.

### Phenotypic correlation of GFD and other traits

**Fig. 1:**
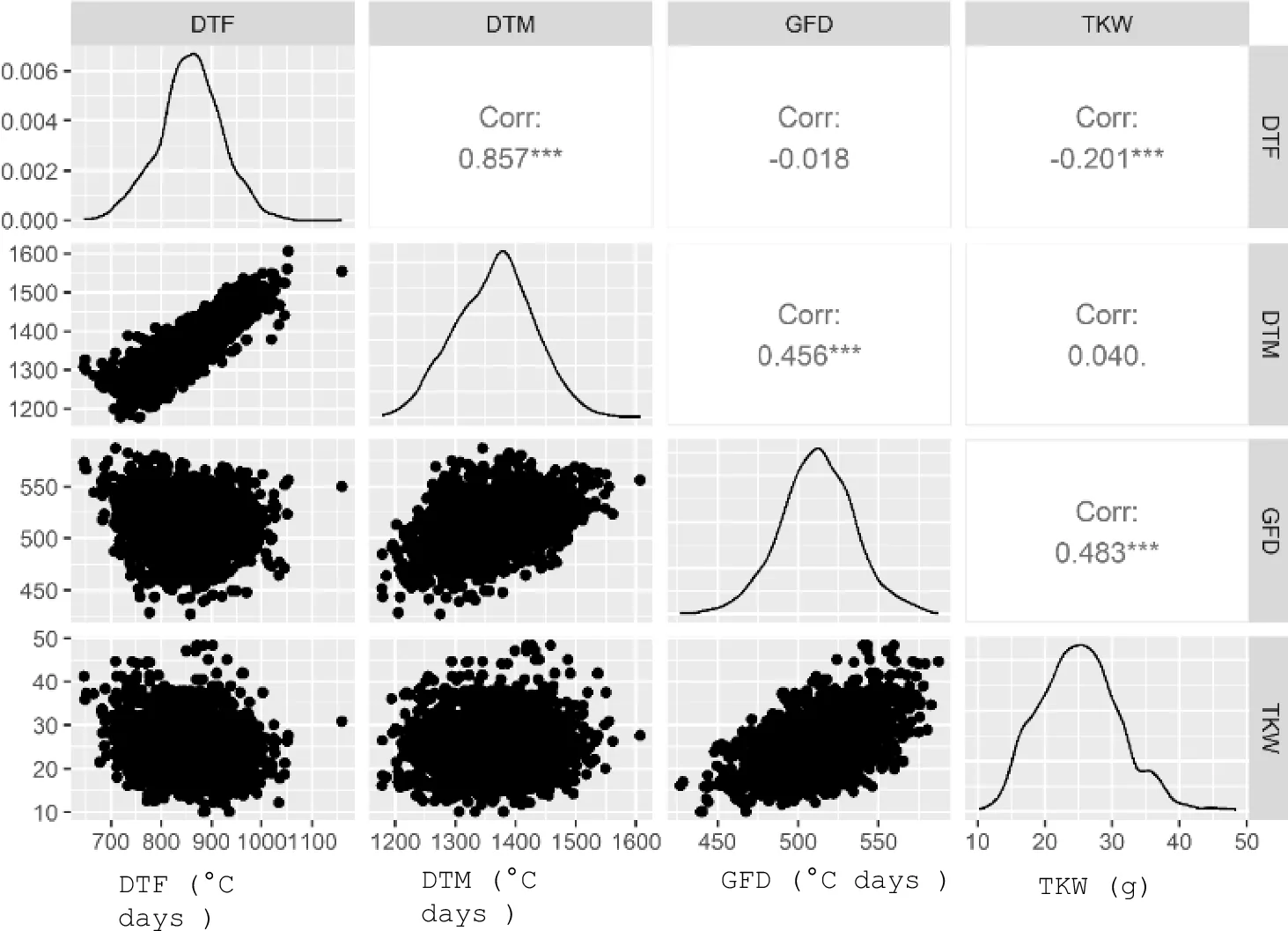
Pearson’s correlation for Days to 50% flowering (DTF), Duration to maturity (DTM), Grain filling duration (GFD), and Thousand kernel weight (TKW) from HRF2 data (BLUEs) showing the association between GFD and other yield related traits in grain sorghum

A Pearson’s correlation analysis for GFD and yield related traits revealed that GFD was significantly positively correlated with DTM and TKW but had a non-significant negative correlation with DTF. DTF was significantly negatively correlated with TKW while DTM had a non-significant low correlation with TKW (Fig. 1).

### Marker trait associations for GFD in the sorghum diversity panel

**Fig. 2:**
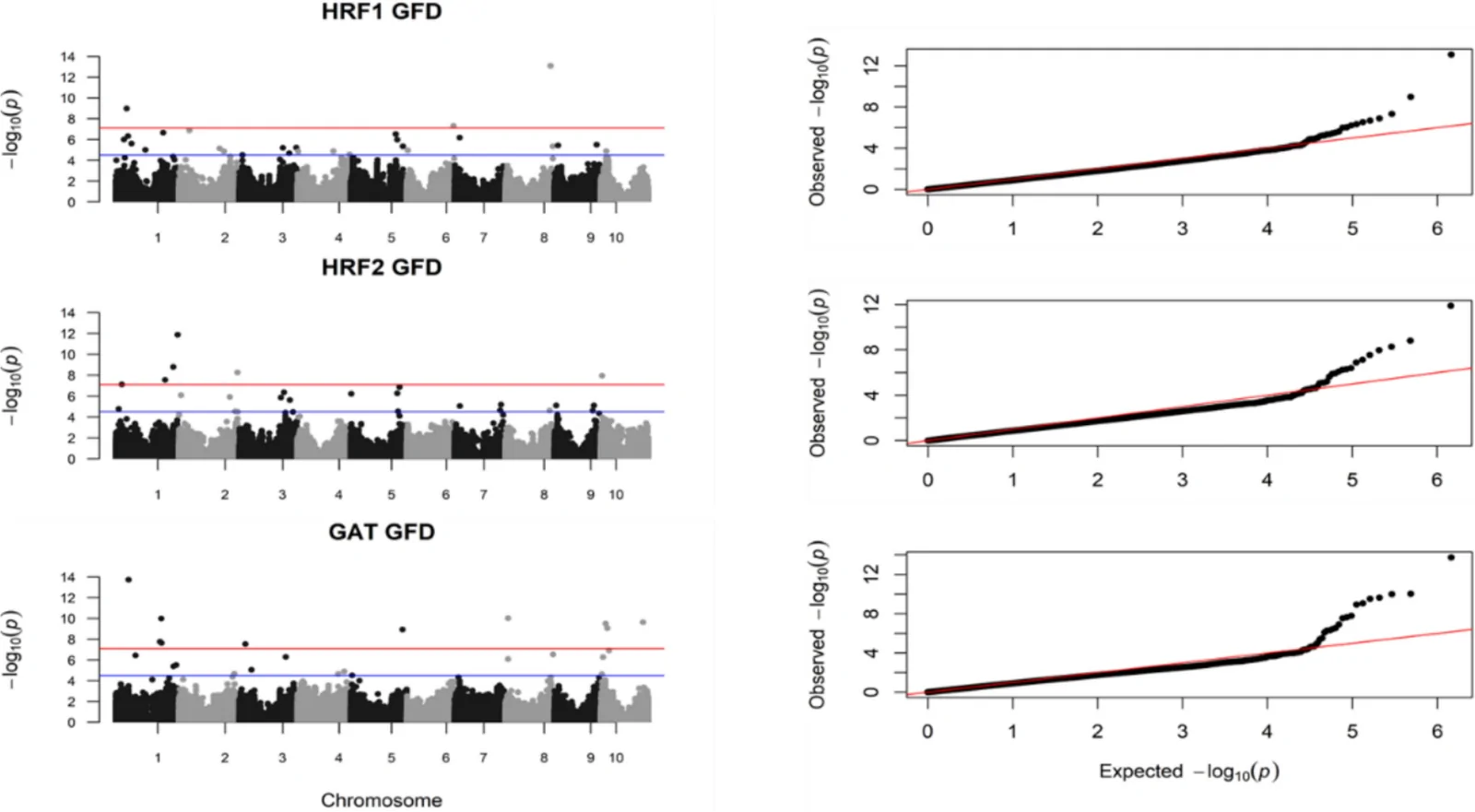
Manhattan plots and QQ plots for grain filling duration (GFD) trait across the locations HFR1, HRF2 and GAT. Points above the red line (P-value 1.53e−07) on the Manhattan plot represent significant genomic regions while points above the blue horizontal line (P-value 9.79e−05) and below the red horizontal line represent suggestive genomic regions associated with grain filling duration. The corresponding QQ plots show the model fitness

**Fig. 3:**
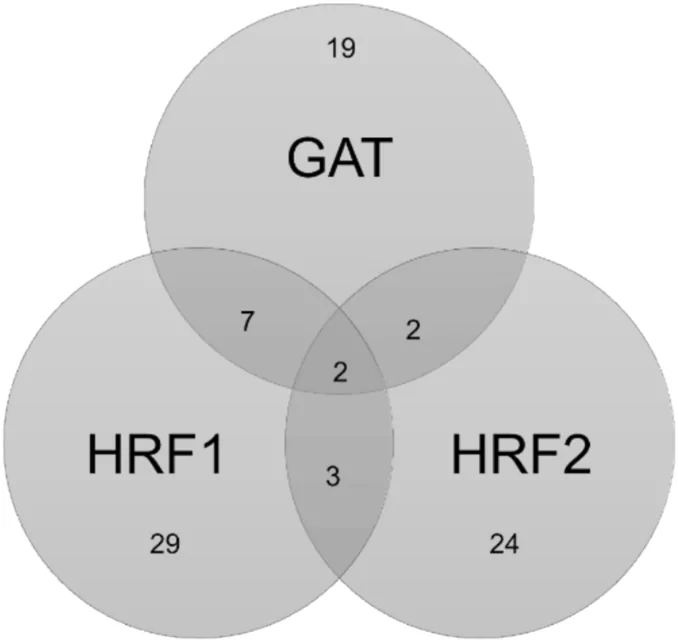
Venn diagram showing unique and common QTLs across the three environments GAT, HRF1 and HRF2. Numbers within the outer parts of circles denote unique QTLs per environment while numbers in the intersection of the circles denote common QTLs between those environments

GWAS analyses for GFD conducted independently for each of the three experiments at HRF1, HRF2 and GAT identified a total of 117 significant and suggestive marker trait associations/SNPs at a significance P-value < 9.79e−05. The highest number of significant SNPs was identified from the HRF1trial (49 in total), while HRF2 and GAT identified 34 and 35 SNPs respectively. One SNP on chromosome five was significant at both HRF1 and HRF2. Overall, SNPs were distributed throughout all the chromosomes with chromosome one having the most and chromosome six the least number of identified SNPs (Fig. 2 Supplementary Table 3). For onward analysis, the 117 significant and suggestive SNPs were clustered into 86 unique QTLs based on a 1 cM window around each SNP as previously described in (Tao et al. 2020) for the sorghum diversity panel. The 86 QTL regions were distributed throughout the 10 chromosomes, with HRF1, having 29, HRF2 24 and GAT 19 unique QTLs respectively. 14 QTLs were common in at least two environments, two of which were found common across all the three environments (Fig. 3). The two QTLs identified across all the three environments were QGFD 1.6 on chromosome one and QGFD 7.1 on chromosome seven. These regions have previously been identified in different studies and are associated with grain traits including thousand kernel weight, grain length, grain width, grain volume and grain thickness (Tao et al. 2021), and sessile spikelet width (Takanashi et al. 2021). Additionally, the QQ plots from the respective GWAS across the environments showed good model fit with no inflation in the observations (Fig. 2).

### Single marker analysis revealed common genomic regions for GFD and TKW

**Fig. 4:**
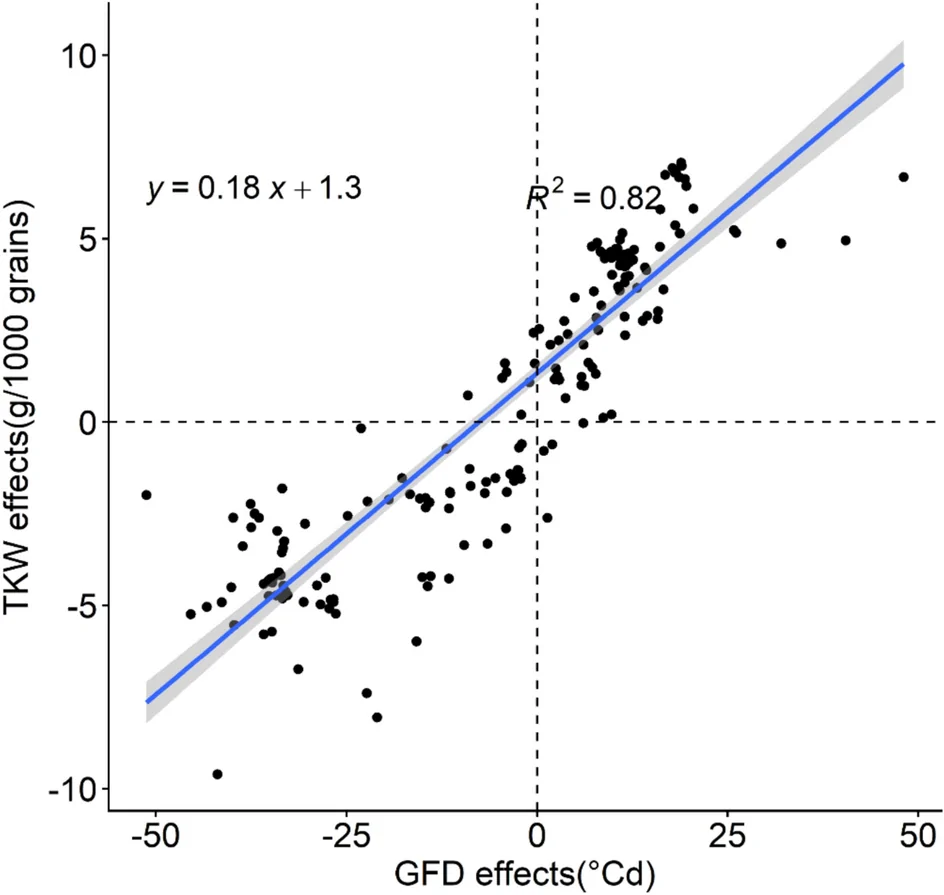
Marker level correspondence of Grain filling duration (GFD) SNP effects and Thousand kernel weight (TKW) SNP effects when TKW trait is tested for association with GFD markers in a single marker analysis. The equation describes the relationship and the R-Squared value the magnitude of the relationship. The broken vertical and horizontal line through the origin is to show the direction of the SNP effects

All effective SNPs identified in the initial GWAS analysis for GFD were subsequently tested in a single-marker analysis and found to be strongly associated with GFD, with a P-value of <0.0001. Similarly, when these SNPs were tested for association with TKW all but four were highly significantly associated at P-value <0.0001, and all but one were significant at P-value <0.05. The individual SNP effects for GFD when compared to those of TKW showed that the SNPs affected the two traits behaved in a similar fashion in terms of both the effect size and effect. The GFD SNP effects could explain up to 82% of the observed variation in the TKW SNPs effects. The largest individual SNP effect for GFD accounted for a difference of 51-degree days in GFD, equivalent to three diurnal days at ambient temperature, while the smallest SNP effect accounted for a 0.3-degree day difference in GFD. A similar trend was observed for TKW with the largest SNP effect accounting for a 10-g difference per 1000 seeds while the smallest accounting for a 0.03-g difference per 1000 seeds (Fig. 4).

### Coincidence of GFD QTLs with expressed genes between pollination and maturity

A comparison between the 86 GFD QTLs with 938 genes exclusively expressed at each respective period from 1–2 days before pollination to physiological maturity in grain sorghum (Jain et al. 2024) revealed 59 QTLs were in linkage disequilibrium (LD) with 167 of these genes. A Chi square test (*X*^2^ test) revealed a significant enrichment of the expressed genes for GFD QTLs at P< 0.0001. Further, 25,49,10,7 and 5 QTLs each mapped to genes expressed 1–2 days before pollination (1–2 DBP), 0–2 days after pollination (0–2 DAP), 10 days after pollination (10 DAP), 20 days after pollination (20 DAP) and 30 days after pollination (30 DAP) respectively (Supplementary Table 2). 77% of these QTLs mapped with genes expressed exclusively early in the pre and post pollination phase of seed development (1–2 DBP and 0–2 DAP). These observations suggest that GFD could possibly be determined by plant growth and development happening early before flowering. The 27 QTLs not specifically mapped to the genes expressed exclusively in the stages above, could be commonly expressed in all the stages from pollination to maturity as well as before pollination.

### Collocation of GFD QTLs with candidate genes for grain size, starch and protein content in sorghum

A comparison of GFD QTLs to 185 sorghum candidate genes associated with grain size, starch and protein content as summarised in (Jain et al. 2024; Tao et al. 2017) revealed that ~53% GFD QTLs (46 of 86) were in LD with at least one of these candidate genes, a significant enrichment at P<0.0001 *X*^2^ test). 42 of the 54 candidate genes in LD with GFD QTLs were associated with grain size, 9 with starch content and 3 with protein content (Jain et al. 2024) (Table 1). The candidate genes associated with grain size were functional in regulating cell proliferation, elongation and division, as well as phytohormone mediated regulation of grain size (Jain et al. 2024). The QTLs QGFD1.13, QGFD2.11, QGFD7.1, QGFD7.4 and QGFD10.4 were within 0.2–0.6 cM of the candidate genes *Sobic.001G485400, Sobic.001G481400, Sobic.002G367300, Sobic.007G193500, Sobic.007G054700* and *Sobic.010G110100* respectively. The rice orthologues of these candidate genes except for *Sobic.010G110100* have been shown to be involved positively in cell proliferation, elongation with resultant increases in grain width, grain size and grain filling (Liu et al. 2015; Lo et al. 2020; Wang et al. 2012, 2015a, 2015b).The rice orthologue of *Sobic.010G110100* suppresses cell proliferation and negatively regulate grain size and weight (Hao et al. 2021). Additionally, these candidate genes have been shown to have peak expression early in the prepollination, post fertilisation and early grain filling in sorghum (Jain et al. 2024) indicating their potential role in early embryogenesis and endosperm development revealing that grain filling could be determined early in the panicle development phases. Candidate genes associated with phytohormone signalling were collocated within 0.2–1.4 cM of QTLs QGFD1.9, QGFD1.11, QGFD1.13,QGFD1.17,QGFD3.2, QGFD4.6 and QGFD10.4 corresponding to *Sobic.001G172400,Sobic.001G120900, Sobic.001G488500, Sobic.001G109100, Sobic.003G257400, Sobic.004G237000* and *Sobic.010G111200*. The rice orthologue of *Sobic.001G120900* is a negative regulator of grain size through a reduction of gibberellic acid (GA) signalling (Lan et al. 2020) while mutants of the rice orthologue of *Sobic.010G111200* show reduced GA and decreased grain weight and width (Shi et al. 2020). *Sobic.001G109100*, *Sobic.001G172400* and *Sobic.004G237000* have orthologues in rice that are involved in the brassinosteroid (BR) pathway signalling to reduce grain size and increase cell proliferation, expansion and grain length respectively. *Sobic.001G488500* and *Sobic.003G257400* were positive regulators of grain size through ethylene mediated reduction in cell proliferation with resultant increases in grain length and cell size in rice spikelets (Chen et al. 2013) and cytokinin (CK) mediated regulation of grain size respectively (Xiao et al. 2019; Yin et al. 2020) (Table 1).

**Table 1:** Concurrence of grain sorghum candidate genes with GFD QTLs. Corresponding genes in rice, maize and Arabidopsis have been provided with their predicted functions. The start and end predicted physical genetic and cM position for the candidate gene is provided. The cM distance from QTL position to the candidate gene is also provided as (cM from QTL*)*. The sorghum consensus map (Mace et al. 2009) was used for the genetic and physical distances

Gene ID	Gene name	Rice/Maize/Arabidopsis orthologue	cM	LG	START	END	cM from QTL	GFD QTL	Function	Reference
Sobic.001G056700	*O2*	Zm00001d018971	10.94	1	4,275,459	4,279,430	0.16	QGFD1.18	Regulatory protein opaque−2	(Hartings et al. 1989)
Sobic.001G107100	*SRS5/TID1*	LOC_Os11g14220	23.73	1	8,265,620	8,268,721	1.02	QGFD1.17	Induces cell elongation in spikelet cells and produces longer grains	(Segami et al. 2012) (Sunohara et al. 2009) (Segami et al. 2017)
Sobic.004G245000	*AHK4*	At2g01830	107.54	4	59,266,365	59,273,133	0.13	QGFD4.6	CHASE domain containing histidine kinase protein	Riefler et al. (2005)
Sobic.001G172400	*BRD1*	LOC_Os03g40540	36.53	1	14,434,718	14,438,560	0.63	QGFD1.9	BRD1 encoded protein catalyses the C‐6 oxidation step to produce active BR which increases cell proliferation and expansion in grains	(Hong et al. 2002) (Mori et al. 2002)
Sobic.004G214100	*BC14*	Os02g0614100	104.77	4	56,389,819	56,395,087	1.44	QGFD4.6	Golgi-localized nucleotide sugar transporters	Zhang et al. (2011)
Sobic.002G367600	*BG2*	Os07g0603700	179.20	2	72,744,933	72,746,890	0.70	QGFD2.11	Cytochrome P450	Xu et al. (2015)
Sobic.008G152800	*CBL3*	AT4G26570	139.82	8	58,517,448	58,523,171	0.59	QGFD8.3	calcineurin B-like 3	Eckert et al. (2014)
Sobic.002G272700	*EOD3/CYP78A6*	At2g46660	147.07	2	65,585,643	65,588,410	0.07	QGFD2.7	oxygen binding	Fang et al. (2012)
Sobic.001G184900	*Expressed protein*		40.28	1	15,806,732	15,807,894	0.06	QGFD1.1		
Sobic.001G341700	*GS3/zmGS3*	Os03G0407400	130.78	1	62,910,779	62,916,258	1.03	QGFD1.16	qTGW1a encodes a G‐protein subunit which negatively regulates grain size	Zou et al. (2020)
Sobic.001G485400	*BG1*	LOC_Os03g07920	188.47	1	75,624,275	75,627,243	0.39	QGFD1.13	Overexpression positively regulates grain size due to increased cell proliferation	(Liu et al. 2015; Lo et al. 2020)
Sobic.010G047400	*HGW*	Os06g0160400	32.75	10	3,668,202	3,673,695	0.40	QGFD10.5	ubiquitin-associated domain protein	
Sobic.001G488500	*OsFBK12*	LOC_Os03g07530	189.02	1	75,844,162	75,849,593	0.94	QGFD1.13	Acts as repressor for downstream gene *SAMS1*and reduces ethylene level and cell proliferation but increases grain length by increasing cell size in spikelet hull	Chen et al. (2013)
Sobic.002G056000	*MET1*	At5G49160	22.90	2	5,374,690	5,386,770	0.68	QGFD2.3	methyltransferase 1	Xiao et al. (2006)
Sobic.002G054800	*O2*	Zm00001d018971	22.63	2	5,243,140	5,247,362	0.95	QGFD2.3		
Sobic.001G254200	*OsFBK12*	Os03g0171600	55.24	1	28,465,427	28,472,547	0.12	QGFD1.4		Chen et al. 2013
Sobic.002G367300	*qGW7/GL7*	LOC_Os07g41200	179.12	2	72,705,386	72,710,986	0.62	QGFD2.11	Encodes a TONNEAU1‐recruiting motif protein which enhances cell elongation resulting larger grains with improved quality	Wang et al. (2015a)
Sobic.008G173900	*OsPPKL3*	Os12g0617900	145.36	8	60,836,806	60,845,885	0.35	QGFD8.2	Extra-large grain	Zhang et al. (2012)
Sobic.001G254100	*PGL1*	Os03g0171300	55.23	1	28,215,025	28,215,913	0.10	QGFD1.4		Heang and Sassa (2012a)
Sobic.001G488400	*PGL1*	Os03g0171300	188.98	1	75,826,748	75,828,379	0.89	QGFD1.13		Heang and Sassa (2012a)
Sobic.010G091700	*PGL2*	Os02g0747900	44.86	10	8,099,384	8,100,834	1.99	QGFD10.3		Heang and Sassa (2012b)
Sobic.004G237000	*PGL2/BUL1*	LOC_Os02g51320	107.18	4	58,488,864	58,490,438	0.48	QGFD4.6	In BR biosynthesis pathway, *BUL1* upregulates *BDG1* which increases grain length	Heang and Sassa (2012b) Jang and Li (2017)
Sobic.001G468400	*Prol1.1*	Zm00001d028129	184.76	1	74,135,478	74,137,316	0.33	QGFD1.6		Wills et al. (2013)
Sobic.004G247000	*Gln-4*	Zm00001d051804	107.64	4	59,472,640	59,476,805	0.03	QGFD4.6	Glutamine synthetase isoenzymes	Martin et al. (2006)
Sobic.007G193500	*SPL16/qGW8*	LOC_Os08g41940	130.54	7	62,605,971	62,612,183	0.25	QGFD7.4	Enhances cell proliferation which increases grain width and grain filling	Wang et al. (2012) Wang et al. (2015a)
Sobic.001G484200	*RGA1/D1*	Os05g0333200	188.23	1	75,526,130	75,530,742	0.14	QGFD1.13		Ashikari et al. (1999)
Sobic.003G380900	*SERF1*	Os05g0420300	146.25	3	69,444,094	69,445,096	1.97	QGFD3.11		Schmidt et al. 2014
Sobic.009G141500	*SERF1*	Os05g0420300	87.26	9	49,879,082	49,879,738	0.03	QGFD9.6		Schmidt et al. (2014)
Sobic.009G049400	*SRS3*	Os05g0154700	54.37	9	4,902,297	4,908,926	0.64	QGFD9.3	Reduces grain length	Kitagawa et al. (2010)
Sobic.001G170800	*Transport protein*	-	36.26	1	14,278,553	14,283,299	0.36	QGFD1.9		
Sobic.004G133600	*ZmSWEET4c*	Zm00001d015912	71.54	4	21,285,737	21,291,316	1.65	QGFD4.3		Sosso et al. (2015)
Sobic.010G110100	*bZIP47*	LOC_Os06g15480	55.75	10	11,004,657	11,007,712	0.23	QGFD10.4	Suppresses cell proliferation and regulates grain size and weight negatively	Hao et al. (2021)
Sobic.010G111200	*GSR1/GW6/* *GASR7*	LOC_Os06g15620	55.80	10	11,197,868	11,198,820	0.27	QGFD10.4	Mutants show reduced GA content and decreased grain width and weight	Shi et al. (2020)
Sobic.001G482600	*TIFY 11b*	Os03g0181100	187.95	1	75,415,876	75,416,893	0.13	QGFD1.13	TIFY gene	Hakata et al. (2012)
Sobic.001G101700	*GIF1*	LOC_Os03g52320	22.82	1	7,782,002	7,785,807	0.11	QGFD1.17	Transcriptional cofactor *GIF1* interacts with *GRF4* and enhance grain size	Li et al. (2016),
Sobic.001G109100	*DLT2*	LOC_Os03g51330	24.12	1	8,469,856	8,473,335	1.41	QGFD1.17	A GRAS‐family member enhances transcriptional activity of *DLT2‐DLT‐BZR1* complex to modulate BR pathway signalling reducing grain size	Zou et al. (2023)
Sobic.001G120900	*SLR1*	LOC_Os03g49990	25.32	1	9,381,697	9,384,098	1.19	QGFD1.11	SLR1 negatively affects grain size via GA signalling	Lan et al. (2020)
Sobic.001G455900	*MADS1/qLGY3*	LOC_Os03g11614	182.92	1	73,188,105	73,199,446	0.42	QGFD1.6	Encodes MADS1 TF which interacts with DEP1 and affects grain size	Liu et al. (2018), Yu et al. (2018)
Sobic.001G481400	*LG3*	LOC_Os03g08470	187.70	1	75,312,217	75,313,912	0.39	QGFD1.13	Encodes TF which facilitates cell elongation and increases grain size	Yu et al. (2018)
Sobic.002G192600	*NAC20/26*	LOC_Os01g01470	93.48	2	57,932,635	57,934,018	0.24	QGFD2.6	It positively regulates the genes involved in starch and storage protein biosynthesis	Wang et al. (2020a), Chen et al. (2020)
Sobic.002G360900	*GASR9*	LOC_Os07g40240	178.27	2	72,279,450	72,280,318	0.23	QGFD2.11	Encodes a Gibberellic acid‐stimulated transcript (GAST) family protein which facilitates cell elongation resulting in longer grains	Li et al. (2019)
Sobic.002G271200	*UGE3*	LOC_Os09g35800	146.80	2	65,474,918	65,477,505	0.34	QGFD2.7	Shows UDP‐galactose/glucose epimerase activity that facilitates substrates for polymerization of polysaccharides	Tang et al. (2022)
Sobic.002G054400	*PK2/PKpα1*	LOC_Os07g08340	22.58	2	5,219,182	5,223,489	1.00	QGFD2.3	Encodes plastidic pyruvate kinase which takes part in biosynthesis of starch in endosperm, formation of compound granule and grain filling	Cai et al. (2018)
Sobic.003G257400	*BG3/PUP4*	LOC_Os01g48800	109.98	3	59,557,217	59,558,392	0.79	QGFD3.2	Encodes purine permease which maintains cytokinin distribution and positively regulates grain size	Xiao et al. (2019) and Yin et al. (2020)
Sobic.003G376000	*AAP6*	LOC_Os01g65670	144.20	3	69,059,581	69,065,077	0.08	QGFD3.11	Positively regulates seed protein content and quality	Peng et al. (2014)
Sobic.003G213800	*SBEIII*	LOC_Os06g26234	69.87	3	54,790,313	54,793,810	1.68	QGFD3.10	Involved in upregulation of starch metabolism pathway and starch biosynthesis	Kang et al. (2013)
Sobic.003G230500	*Sh2/APL2*	LOC_Os01g44220	87.20	3	57,000,119	57,007,815	1.01	QGFD3.9	Encodes large subunit of ADP‐glucose pyrophosphorylase. It acts as starch biosynthetic enzyme which suppresses starch biosynthesis pathway	Hannah and Nelson (1976)
Sobic.004G238600	*SBEIII*	LOC_Os02g51070	107.25	4	58,642,327	58,646,857	0.42	QGFD4.6	Involved in upregulation of starch metabolism pathway and starch biosynthesis	Kang et al. (2013)
Sobic.004G256800	*AAP10*	LOC_Os02g49060	116.48	4	60,268,993	60,273,180	0.00	QGFD4.1	Encodes amino acid permease which loads amino acid in endosperm	Wang et al. (2020b), Yang et al. (2020)
Sobic.007G051700	*ASP1*	LOC_Os08g06480	58.48	7	5,282,384	5,291,841	0.09	QGFD7.1	Encodes a transcriptional co‐repressor which affecting branching and spikelet development reducing grain size	Yoshida et al. (2012)
Sobic.007G054700	*NF‐YC10*	LOC_Os01g24460	58.66	7	5,540,371	5,542,244	0.27	QGFD7.1	Positively regulates cell division in spikelet hull cells and endosperm increasing grain width, and grain weight	Jia et al. (2019)
Sobic.010G022600	*Wx*	LOC_Os06g04200	26.92	10	1,860,964	1,865,278	0.07	QGFD10.6	Encodes for granule‐bound starch synthase (GBSS) with a role in amylose biosynthesis in rice endosperm	Yang et al. (2021); Zhang et al. (2021b)
Sobic.010G047700	*SSI*	LOC_Os06g06560	32.77	10	3,694,261	3,702,940	0.42	QGFD10.5	Catalyses formation of amylopectin from ADP‐glucose and upregulates other enzymes involved in starch biosynthesis in endosperm	Fujita et al. (2006)
Sobic.010G072300	*Sh1*	LOC_Os06g09450	35.29	10	5,859,073	5,867,276	1.63	QGFD10.2	Downregulation of *Sh1* leads to lower production of starch. Involved in sucrose synthesis and metabolism	Chourey and Nelson (1976)

### *SbGS3* potentially has a role in moderating GFD in sorghum

**Fig. 5:**
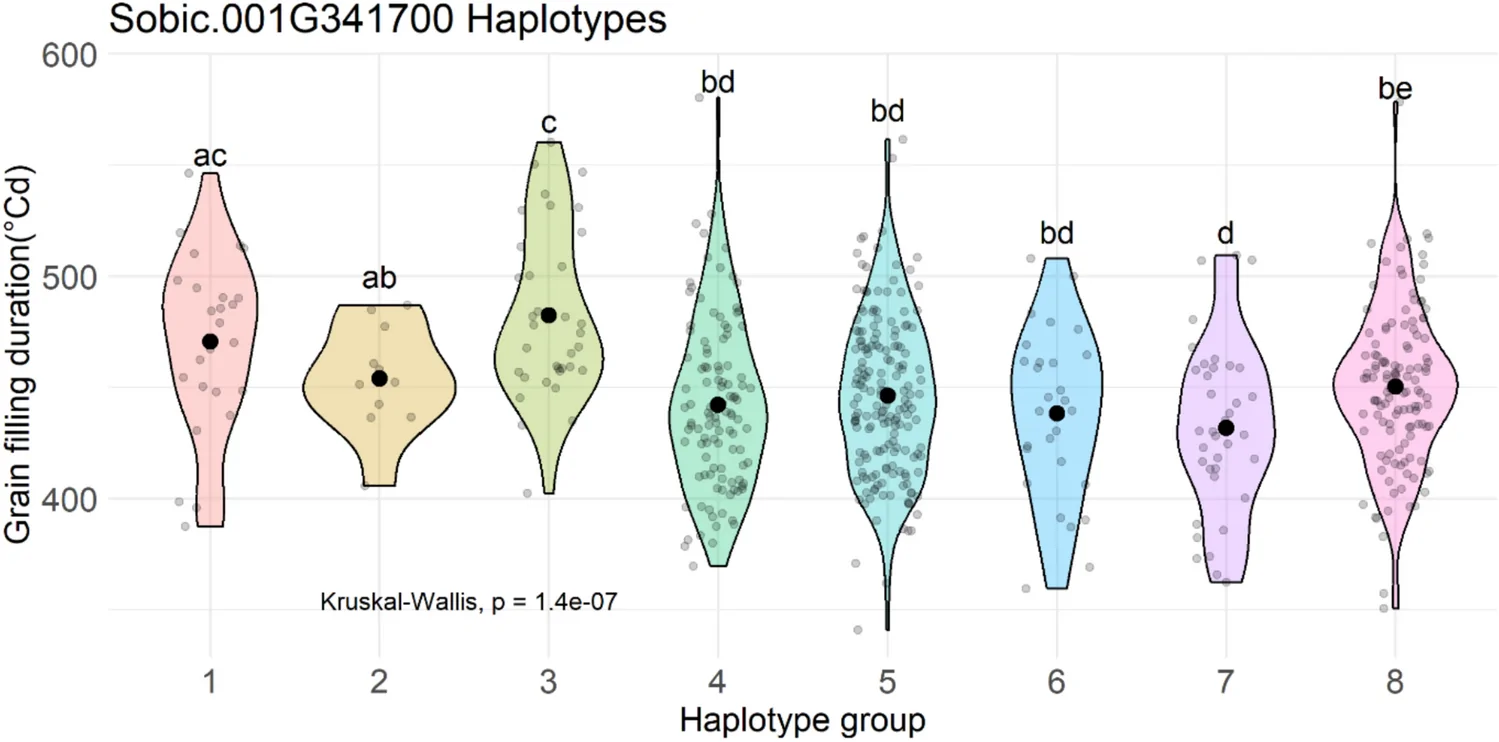
Grain filling duration (GFD) across haplotype groups for *Sobic.001G341700* gene in sorghum. Kruskal–Wallis P-value is presented for global comparisons and Wilcoxon tests significance presented as alphabetical letters above the violin plots for differences in mean between haplotype groups at P<=0.05. Similar letters indicate no significant differences while different letters indicate significant differences

Haplotype analysis for *Sobic.001G341700* (*SbGS3)* a putative orthologue of GS3 in rice that is thought to function as a negative regulator of grain size (Zou et al. 2020), revealed eight different haplotype groups. A pairwise comparison of the individual haplotype groups showed that haplotype 1 had a similar effect on GFD as haplotypes 2 and 3, while haplotype 2 had similar effect on GFD as haplotypes 4,5,6 and 8. Haplotype 7 had similar effects on GFD as haplotypes 4,5 and 6 (Fig. 5). Globally the haplotypes groups were significantly different from each other as shown by the Kruskal–Wallis test P-value. Interestingly, when the haplotype groups were classified based on the sorghum racial groups, the distribution of the haplotypes were more defined. While there were no significant differences within the race for the haplotype groups represented, some key haplotype groups were only present in specific sorghum races. Haplotypes 4,5 and 6 were common across all the racial groups, except for Asian Durra, while haplotype 7 was represented in Asian Durra, Caudatum and Kafir racial groups. Interestingly, haplotype 1 was only present in Asian Durra and Guinea, and haplotype 3 was only present in the Guinea race. Haplotypes 1 and 3 had longer GFD in comparison to the rest of the haplotypes and should be explored further to establish their association with *SbGS3*. The lead SNP tagging the *SbGS3* had a minimum allele frequency (MAF) of 0.43 and an allele substitution effect (β) of − 0.343. Finally, no specific racial group had all the haplotype groups represented.

## Discussion

Increasing cereal crop productivity in challenging production environments caused by the effects of a changing climate remains a high priority for cereal breeders. To date increases in yield have mainly been achieved by increases in grain number (Boyles et al. 2016; Khush 1995; Otwani et al. 2024; Russell 1991) which appears to have greater variability across cereals (Sadras 2007). However there does appear to be variation in grain size that could be exploited to increase yield (Fernández et al. 2022; Xing et al. 2023), studies exploring grain size related traits like GFD that could contribute to increases in grain sizes are lacking. Further, studies on the genetic underpinning of GFD and its contribution to grain size are scarce. To our knowledge, no studies are available that explore the genetic control of GFD in sorghum. This study is the first and largest of its kind to explore the genomic regions associated with GFD in sorghum. We report considerable exploitable genetic variation for GFD, its association with grain size at both phenotypic and genetic levels and propose some candidate genes for GFD in grain sorghum.

### Variation for GFD in grain sorghum

The phenotypic distribution of GFD revealed a wide range of GFD across the tested genotypes and environments, suggesting that GFD is a quantitative trait controlled by multiple loci. The moderate broad sense heritability too shows that GFD could be utilised as a potential useful trait in breeding programmes. Despite accounting for the effects of temperature in the estimation of GFD, the moderate genetic correlation between HRF1 and GAT and HRF2 and GAT environments reveal that there could be other factors, genetic or environmental that influence the estimates of GFD in the tested genotypes. First, could be the contribution of other environmental factors like radiation that was not accounted for in these experiments. Another plausible reason would be the possibility that the tested genotypes could potentially have different cardinal temperature requirements for the grain filling phase as has been suggested by (Tirfessa et al. 2020) in sorghum. Further, the significant positive correlation between GFD, DTM and TKW in these diverse genotypes show that there is opportunity to manipulate GFD without penalty to DTM windows or TKW. DTM stability is crucial in many breeding programmes as it dictates choices for growers to target suitable varieties for specific season lengths. The positive association with TKW implies that increased grain sizes could be attained by increasing the GFD. Additionally, the lack of association of GFD with DTF, is important to guide decisions for possible trait introgression strategies. Elite breeding lines could benefit from introgression of longer GFD attribute with little or no penalty to the desired flowering window, making the trait attractive to breeders. Since many commercial sorghum breeding programmes rely almost entirely on the Kafir/Caudatum crosses (Otwani et al. 2024) due to limitations imposed by the cytoplasmic male sterility system used (Jordan et al. 2011; Reddy et al. 2007), wide hybridisation across all the sorghum racial groups (Weltzien et al. 2006) would provide new opportunities for yield improvement. Targeted crosses including genotypes from the Guinea race that showed consistent longer GFD and are reported to have large grain sizes (Sapkota 2021; Tao et al. 2020) would be a good starting point.

### GFD is closely linked to grain size in sorghum

Single marker analysis of the GFD SNPs, using TKW trait showed high fidelity and correspondence of these SNPs for TKW, suggesting that GFD in highly associated with TKW in the tested genotypes. Additionally, the association of GFD QTLs with candidate genes identified for grain size in sorghum (Jain et al. 2024; Tao et al. 2017) reinforces these observations. 30% of the candidate genes for grain size were in LD with 50% of the GFD QTLs. These candidate genes included a validated gene in sorghum *Sobic.001G341700*, whose QTL, qTGW1a encodes a G-protein subunit negatively regulating grain size (Zou et al. 2020). Haplotype analysis of GFD for this candidate gene revealed that sorghum racial group Guinea, known to have large seed sizes (Sapkota 2021; Tao et al. 2020) and longer GFD had a unique haplotype not present in all the other racial groups corroborating the possible close link between GFD and TKW. Another candidate gene, *Sobic.007G193500* whose rice orthologue *LOC_Os08g41940*, encodes *SPL16/qGW8* gene which is indicated to enhance cell proliferation increasing grain width and grain filling (Wang et al. 2012, 2015a) was also enriched within the GFD QTLs. Previous studies in sorghum (Yang et al. 2009), maize (Fernández et al. 2022; Xing et al. 2023), rice (Wang et al. 2008; Yang et al. 2008), wheat (Chapman et al. 2021; Xie et al. 2015) and barley (Radchuk et al. 2021) discuss potential of utilising grain filling dynamics for yield improvement. While the association of GFD and TKW were considerably modest at the phenotype level, the association at the marker level were very robust. These observations could be potentially due to various factors; first, the environmental variations in temperature along with Genotype by Environment (GxE) interactions could have caused higher phenotypic variance across environments reducing the phenotypic correlation. While expressing GFD in thermal units should potentially account for temperature differences across environments, genotypic differences in basal temperature requirements have been observed previously in sorghum (Tirfessa et al. 2020). Second, from quantitative genetics standpoint, the portions of genetic variances captured at the phenotype and marker levels differ. At the phenotype levels, both the additive and non-additive genetic variances are reflected in the association while only predominantly additive genetic variance is captured at the marker level (Lynch and Walsh 1998) which could explain the observed differences. The observed links, however, of GFD and TKW both at phenotype and genomic levels provide opportunities to explore further these observations. For instance, it remains unknown if GFD is fundamentally a part of grain size biology or an independent phenomenon acting in concert with grain size, while it may be plausible to infer pleiotropy in the association of GFD and TKW, tight linkage of genes controlling the two traits independently cannot be ruled out and should be further investigated. Since both traits are complex and appear to interact, further studies are warranted to understand these traits better, especially regarding TKW, whose study and interpretation is limited in the current study as it was only recorded in one environment.

### GFD is dynamic and determined by mechanisms happening both before and after anthesis in sorghum

GFD is estimated from flowering to maturity in many crop species including sorghum (Gambín and Borrás 2012). The in silico observation that 77% of expressed genes that were in LD with GFD QTLs were exclusively expressed in the early pre anthesis and post anthesis period reveal that determination of GFD like many panicle and grain associated traits (Van Oosterom and Hammer 2008) happen before anthesis. Most of these candidate genes were associated with the regulation of cell proliferation and elongation through hormone mediated pathways in rice. The rice orthologue of *Sobic.010G110100, LOC_Os06g15480* encodes a gene *bZIP47* which suppresses cell proliferation early in the pre anthesis phase in rice negatively affecting grain size and weight (Hao et al. 2021).

Looking at the temporal expression profiles of the enriched candidate genes within the GFD QTLs revealed that these genes could potentially be clustered into three broad groups. First are candidate genes expressed early pre and post anthesis and appear to mediate cell division, elongation and proliferation and eventually determine the cell size and number of the panicle and floral organs (Liu et al. 2015; Lo et al. 2020; Yan et al. 2024). Similar observations of pre anthesis organ size in sorghum (Takanashi et al. 2021; Yang et al. 2009) has been associated with grain size and GFD. Secondly are genes associated with phytohormone mediated regulation of cell size, cell number and GFD. The sorghum candidate genes *Sobic.001G120900* and *Sobic.010G111200* are orthologous to rice genes *SLR1* (Lan et al. 2020) and *GSR1/GW6/GASR7* (Shi et al. 2020) that reduce grain weight and increase grain width and weight respectively through GA signalling. These candidate genes had peak expression in the early pre anthesis and post anthesis phase in sorghum (Jain et al. 2024). Candidate genes associated with BR signalling *Sobic.004G237000, Sobic.001G172400* and *Sobic.001G109100* orthologous to rice candidate genes *PGL2/BU1, BRD1* and *DLT2* respectively were highly expressed early in the grain filling period 1- 10 days post anthesis. *BU1* has been reported to upregulate *BDG1* to increase grain length in rice (Heang and Sassa 2012b). The rice orthologue of *Sobic.001G488500, LOC_Os03g07530* (Chen et al. 2013) is associated with ethylene mediated grain length increases in the spikelet hull. The high expression of *Sobic.001G488500* during early grain filling in sorghum (Jain et al. 2024) is consistent with the role of ethylene in grain filling, fruit ripening and progression to maturity (Kim et al. 2013; Magar et al. 2024; Patterson and Bleecker 2004; Sexton and Roberts 1982). Candidate gene *LOC_Os01g48800* in rice (Xiao et al. 2019; Yin et al. 2020), orthologous to *Sobic.003G257400*, encodes purine permease maintaining distribution of cytokinin and regulating grain size positively. *Sobic.003G257400* high expression during grain filling suggests its role in grain filling consistent with reports in sorghum on the role of cytokinins in mediating grain filling and grain size (Heiniger et al. 1993), in wheat (Wheeler 1972) and maize (Xu et al. 2024). Finally, are candidate genes associated with starch biosynthesis, metabolism, transport, storage and protein storage. These candidate genes were expressed from early anthesis through to physiological maturity in sorghum (Jain et al. 2024). The sorghum candidate genes *Sobic.003G213800* and *Sobic.004G238600* were highly expressed in early anthesis and start of grain filling. Their rice orthologues, *LOC_Os06g26234* and *LOC_Os02g51070* are involved in starch biosynthesis, metabolism and accumulation in the endosperm (Kang et al. 2013; Li et al. 2018). Starch accumulation has been shown to occur during grain filling in rice (Liu et al. 2024) suggesting that the identified candidate gene could have a role in sorghum grain filling. *Shrunken 1* and *shrunken 2*, identified in maize (Chourey and Nelson 1976; Hannah and Nelson Jr 1976) which are suppressors of starch biosynthesis, were enriched in GFD QTLs, and appear to potentially mediate GFD in sorghum and other cereals. The candidate gene *Sobic.010G022600* corresponding to the *waxy* gene which encodes granule bound starch synthase and mediates amylose biosynthesis in cereals (McIntyre et al. 2008; Zhang et al. 2021a). Overall, these findings show that GFD is a complex trait and potentially determined from early in the panicle development phase in sorghum. These revelations could suggest potential interactions of GFD with grain number determination (Van Oosterom and Hammer 2008) and require further investigations to reveal the nature of such interactions and their relevance in breeding for extended GFD genotypes.

### Deploying extended GFD genotypes in sorghum breeding programmes

The results from the current study highlight plausible strategies for introgression of extended GFD trait into elite sorghum genotypes. First, since commercial hybrid breeding programmes have utilised the Caudatum and Kafir races in most of the current elite lines and hybrids, wide hybridization utilising the other sorghum racial groups, especially Guinea would provide opportunity to develop extended GFD lines. Secondly, since the genomic regions associated with GFD and grain size have been identified, fine gene mapping could be explored in biparental populations to further understand the traits. These genomic regions may also be explored in elite breeding population to identify if indirect selection for extended GFD has happened in the breeding programs. These strategies together would contribute to quantify the value of GFD trait to underpin further resource investment.

## Conclusion

This study established a potential close link between GFD and grain size in grain sorghum, reports candidate genes that potentially mediate GFD and could be targeted for breeding for longer GFD genotypes. The revelation that GFD is potentially determined by mechanisms that occur before anthesis like many other panicle and seed related traits presents opportunity to unravel associations between GFD and grain number among others and explore strategies to use them in breeding programmes. Additionally, a subset of GFD associated candidate genes could be utilised directly as a selection index together with grain size and number to improve selection outcomes in sorghum breeding. Finally, since the current study was limited by one site analysis of TKW, future studies should explore more the association of GFD and TKW in multiple environments and populations to expand the understanding of the association in grain sorghum.

## Supplementary Information

Below is the link to the electronic supplementary material.

Table 1: Model parameters for the linear mixed model used across the sites and for the single marker analysis.

Below is the link to the electronic supplementary material.

Table 2: Sorghum candidate genes their genetic distance and physical distances across the chromosomes and their expression at different stages pre and post anthesis obtained from (Jain et al. 2024). Colocalization with GFD QTLs represented by the cM distance from the expressed gene. DBP - Days before pollination, DAP - Days after pollination. Table 3: Table showing the markers for grain filling duration, their chromosome location (Chr), physical position (Pos), negative log10 value at each site, the genetic distance (cM) the QTL identified and concurrence with identified QTLs from previous studies in the references and traits associated. T - Thousand grain weight, V-Grain volume, L- Grain length, W - Grain width and H - Grain thickness. SSL -Sessile spikelet length ,SSW- Sessile spikelet width ,PEL- Pedicel length, ANL- Anther length, STIW - Stigma width.

## Data Availability

The Phenotypic data is available on DRYAD https://doi.org/10.5061/dryad.hhmgqnkrn The genomic data is available from the corresponding author on reasonable request.
