# Supplementary material for: Common genetic control for grain filling duration and kernel weight in grain sorghum
Source: Theor Appl Genet. 2026 Oct 12;139(11):297. doi: 10.1007/s00122-026-05409-0 (PMC13658289; doi:10.1007/s00122-026-05409-0)
Supplement: Supplementary file 1 [file 122_2026_5409_MOESM1_ESM.docx]

Supplementary Table 1: Model parameters for the linear mixed model used across the sites and for the single marker analysis.

| Site | Fixed terms | Random terms |
| --- | --- | --- |
| Hermitage | Site | corgh(Site):Genotype+diag(Site) :Row +at(Site,1):Column+at(Site,2):spl(lincol) |
| Gatton | Site | corgh(Site):Genotype+diag(Site) :Row  +at(Site,1):Column |
| Single marker  Analysis | SNPs | vm(MGeno, MMT, singG = "PSD") + MGeno |
